# Unsupervised Online Assessment of Visual Working Memory in 4- to 10-Year-Old Children: Array Size Influences Capacity Estimates and Task Performance

**DOI:** 10.3389/fpsyg.2021.692228

**Published:** 2021-08-06

**Authors:** Shannon Ross-Sheehy, Esther Reynolds, Bret Eschman

**Affiliations:** ^1^Department of Psychology, University of Tennessee, Knoxville, Knoxville, TN, United States; ^2^Department of Psychology, Florida International University, Miami, FL, United States

**Keywords:** visual working memory, child development, online assessment, cognitive development, capacity estimates

## Abstract

The events of the COVID-19 Pandemic forced many psychologists to abandon lab-based approaches and embrace online experimental techniques. Although lab-based testing will always be the gold standard of experimental precision, several protocols have evolved to enable *supervised* online testing for paradigms that require direct observation and/or interaction with participants. However, many tasks can be completed online in an *unsupervised* way, reducing reliance on lab-based resources (e.g., personnel and equipment), increasing flexibility for families, and reducing participant anxiety and/or demand characteristics. The current project demonstrates the feasibility and utility of unsupervised online testing by incorporating a classic change-detection task that has been well-validated in previous lab-based research. In addition to serving as proof-of-concept, our results demonstrate that large online samples are quick and easy to acquire, facilitating novel research questions and speeding the dissemination of results. To accomplish this, we assessed visual working memory (VWM) in 4- to 10-year-old children in an unsupervised online change-detection task using arrays of 1–4 colored circles. Maximum capacity (max K) was calculated across the four array sizes for each child, and estimates were found to be on-par with previously published lab-based findings. Importantly, capacity estimates varied markedly across array size, with estimates derived from larger arrays systematically underestimating VWM capacity for our youngest participants. A linear mixed effect analysis (LME) confirmed this observation, revealing significant quadratic trends for 4- through 7-year-old children, with capacity estimates that initially increased with increasing array size and subsequently decreased, often resulting in estimates that were *lower* than those obtained from smaller arrays. Follow-up analyses demonstrated that these regressions may have been based on explicit guessing strategies for array sizes perceived too difficult to attempt for our youngest children. This suggests important interactions between VWM performance, age, and array size, and further suggests estimates such as *optimal array size* might capture both *quantitative* aspects of VWM performance and *qualitative* effects of attentional engagement/disengagement. Overall, findings suggest that unsupervised online testing of VWM produces reasonably good estimates and may afford many benefits over traditional lab-based testing, though efforts must be made to ensure task comprehension and compliance.

## Introduction

Infant research is difficult for many reasons. Access to public records is increasingly restricted, contact information is often unpublished, and in many areas, families and communities are becoming wary of privacy concerns and university sponsored research. In addition, the reality of dual-income families continues to make lab-based testing in the early months and years of life a logistical challenge. Although the gold standard of experimental precision will likely always center around lab-based techniques, changing work and family dynamics necessitates a re-evaluation of the gold-standard approach.

The events of the COVID-19 Pandemic forced many psychologists to abandon lab-based techniques and embrace online experimental approaches. This has been particularly difficult for developmentalists, as many infant and child-based testing techniques rely on looking time or eye-tracking methodologies. Fortunately, many innovative approaches have been developed that allow for live face-to-face testing (i.e., *supervised* testing), including commercial video conferencing options like *Zoom* and *Microsoft Teams*, and homegrown software solutions such as *Lookit* (https://lookit.mit.edu). While these approaches facilitate remote observation of the child engaging in the task, they involve many of the same resources as lab-based work, including dedicated experimenters and observers to run test sessions, and pre-scheduled appointments with families. However, for tasks that can be adapted to rely solely on behavioral responses (key presses, mouse clicks, touch screens, etc.), it is possible to do remote online testing in an *unsupervised* way. We report here results from a large-scale unsupervised online change-detection task assessing visual working memory (VWM) development continuously from 4 to 10 years of age.

There are several practical benefits of conducting unsupervised online research. First, it increases session flexibility, allowing participation at optimal times such as after naps, on a rainy Saturday afternoon, or when network traffic is low. Second, it allows for home-based testing, which in addition to being more convenient for parents and children, may decrease the anxiety and demand characteristics that are inevitably a part of supervised testing procedures. Third, unsupervised at-home testing may allow participation from a wider range of children, both neuro-typical and neuro-atypical, and allows for rapid testing over a broad range of ages.

In addition to these practical advantages, there are a host of scientific benefits that may increase data validity and facilitate novel research questions. For example, this approach reduces the time and resources necessary to acquire large sample sizes, increasing power and replicability for even relatively small effects. This speeds dissemination of research findings, and may facilitate novel findings and theory building. Unsupervised online testing can also be conducted regionally, nationally, or even internationally without regard to time zone constraints. In addition to facilitating epidemiological approaches to the study of development, online testing can improve racial, ethnic, and socioeconomic diversity, something that is profoundly lacking from most lab-based research samples. Although access to computers and internet connections may vary across these diverse populations, it is possible for participants to conduct these tasks using a mobile device or tablet, a friend or family member's computer, or public resources such as school, library or community computer banks. Finally, online testing allows the explicit testing of environment factors such as screen size, stimulus size, and method of response (e.g., mouse, keyboard or touchscreen). These features are often either ignored completely or held constant in lab-based tasks, despite the fact that changes in these simple task features might critically influence performance. This form of apparatus diversity additionally ensures that findings are robust, and context independent.

There are of course some drawbacks to unsupervised online testing, including lack of control (Anwyl-Irvine et al., 2020a) and the possibility of parental interference and/or non-compliance with experimental procedures. All of these can be ameliorated to some extent using tools present in most modern online experimental testing suites (e.g., *Gorilla.sc* and *LabVanced.com*), including ability to collect webcam video and to “calibrate” or scale the stimuli based on the estimated screen size. It is also possible to use *indirect* measures to identify questionable data, such as participants whose response times are either too fast to be plausibly completed by the participant (i.e., parental interference), or to reflect effortful decision and response selection (i.e., random button presses). We incorporated several of these approaches in the current project. However, one of the most challenging and underappreciated aspects of successful online testing, is accurately conveying task instructions to the children and to the parents who function as *ad hoc* experimenters. In contrast to supervised testing approaches, it is impossible to gauge understanding and solicit questions from families during unsupervised testing. Thus, it is critically important that the task be piloted in the lab with the target age demographic, to reveal confusing and problematic aspects of the task instructions. This process also facilitates the development of videos and practice trials that maximally enhance task understanding.

Choice of task is also a key factor. The current project incorporates an unsupervised online testing approach to assess development of VWM, which is quite easily adapted to rely solely on behavioral responses (mouse or keyboard clicks, or touches). This task was chosen, because VWM is an essential visuocognitive ability that shows substantial development over the first several years of life (Ross-sheehy et al., 2003; Gathercole et al., 2004; Oakes et al., 2006; Simmering and Spencer, 2008; Simmering and Perone, 2013; Buss et al., 2018; Ross-Sheehy and Eschman, 2019; Reyes et al., 2020), and developmental profiles have already been established across a range of ages (e.g., Cowan et al., 2005; Simmering, 2012). VWM is an active form of short-term memory, that supports the processing of visual spatial information in service of a task or goal (Luck and Vogel, 1997). Many tasks that support early learning rely heavily on VWM, including visual comparison, categorization, spatial navigation, visual search, object learning, spatial reasoning, and math. Thus, VWM is a critically important component of general cognitive development.

Much research has tied VWM to later academic achievement. For example, Bull (2008) found that VWM performance in preschool predicted math problem solving at 8 years of age. Similarly, others have found that VWM in 7- to 14-year-olds predicted performance on a national curriculum math test (Jarvis and Gathercole, 2003). These basic findings have now been replicated numerous times, with most results demonstrating an important connection between early VWM and later math achievement (Tsubomi and Watanabe, 2017; Giofrè et al., 2018; Allen et al., 2019; Chan and Wong, 2019; Kyttälä et al., 2019; Carr et al., 2020). VWM in adults is related to measures of fluid intelligence (Fukuda et al., 2010), and the development of VWM is distinct from verbal WM (Gathercole and Baddeley, 1993; Jarvis and Gathercole, 2003; Giofrè et al., 2018; Kyttälä et al., 2019) and executive function aspects of WM (Jarvis and Gathercole, 2003; Gathercole et al., 2004). Thus, early and frequent access to online VWM assessment tools could significantly enhance detection and possibly intervention for children at risk of cognitive delay. Although the literature on WM training interventions is mixed, recent ERP work with adults demonstrates hopeful evidence of persistent VWM training benefits (Zhang et al., 2020).

### The Current Project

The goal of the current project is to demonstrate the feasibility and validity of unsupervised online testing approaches in child populations, by incorporating a canonical lab-based change-detection task previously used in infant, child and adult populations (Luck and Vogel, 1997; Cowan et al., 2005; Riggs et al., 2006; Ross-Sheehy and Eschman, 2019). The task was adapted for online testing and was used to assess VWM development from 4 to 10 years of age. Our task incorporated a whole-report change-detection approach, meaning all array items were present both in the sample and test arrays, and the child's job was to determine if anything changed from the sample to the test array. Although many adult change-detection tasks now utilize a single-probe or partial report approach (Rouder et al., 2011), we opted to incorporate the whole-report approach for two reasons: First, pilot studies conducted in our lab suggested that younger children struggled to understand the concurrence between sample and test arrays, and altering test arrays might further disrupt within-trial continuity for our youngest participants. Second, this task has already been used successfully in both infant and adult participants (Ross-Sheehy and Eschman, 2019), facilitating the examination of capacity development from infancy to childhood and beyond.

## Methods

### Participants

Our participant pool was a sample of convenience and included all families of children born in local or neighboring counties who had previously expressed an interest in study participation. All registered families with children between the ages of 4 and 11 years during our 6-month data collection window were contacted via email and invited to participate. Of the 2,949 families contacted, 9.93% agreed to participate, resulting in a sample 297 children (see Table 1 for demographics). Unlike standard lab tasks, data quality could not be assessed until after participation was complete. As a first step, we examined survey responses for each participant. This resulted in the exclusion children due to frustration or inability to understand the task (*n* = 3), diagnosis of developmental delay (*n* = 1) or autism spectrum disorder (*n* = 5), incorrect age (*n* = 1), or completing the task using a mobile phone (*n* = 1). We next assessed general task performance by examining the number trials completed out of 80 possible trials, as well as general performance (hit, miss, correct rejection, and false alarm rates). We excluded children who did not complete at least 3 blocks of trials (*n* = 18, *M*_trials_ = 13, *SD*_trials_ = 3.7), and children who selected only a single response button (*n* = 1). Although several children reported a family history of colorblindness (*n* = 11) an examination of their results revealed typical patterns of responding, so they were retained in the sample. Task engagement for the final sample of 267 subjects was very high, *M*_trials_= 76.67, *SD*_trials_ = 12.81.

**Table 1:** Participant counts and demographics by age (years).

**SES (annual)**												**Race**				**Ethnicity**
**Age**	**N**	**M**	**SD**	**Min**	**Max**	**Female**	** <80 K**	**≥80 K**	**Asian**	**Am. Indian**	**Black**	**Pac. Islander**	**White**	**Mult. Race**	**NA**	**Hispanic**
4	43	4.59	0.25	4.02	4.98	42%	23%	77%	9%	2%	2%	2%	81%	0%	2%	7%
5	56	5.50	0.32	5.01	5.99	53%	20%	80%	7%	2%	5%	0%	86%	0%	0%	5%
6	50	6.42	0.28	6.00	6.98	48%	33%	67%	4%	0%	2%	0%	90%	2%	2%	4%
7	31	7.54	0.25	7.02	7.98	38%	24%	76%	9%	3%	13%	0%	72%	0%	3%	0%
8	32	8.43	0.27	8.00	8.97	38%	17%	83%	6%	6%	3%	0%	78%	3%	3%	0%
9	28	9.45	0.32	9.00	9.99	70%	26%	74%	3%	0%	13%	0%	83%	0%	0%	3%
10	26	10.36	0.30	10.03	10.99	52%	9%	91%	4%	0%	11%	4%	81%	0%	0%	7%

### Stimuli

**Figure 1:**
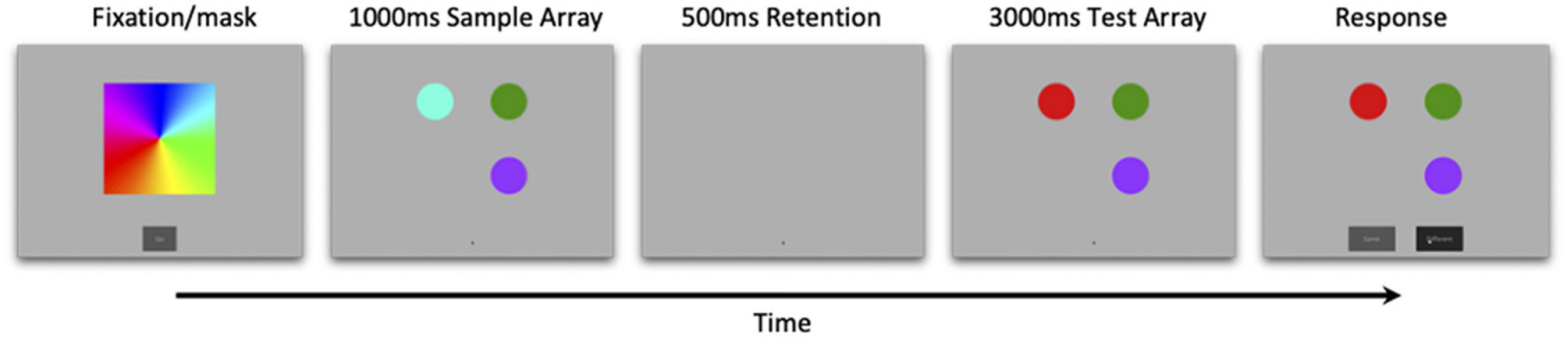
Trial events for online change-detection task. Infants were presented with a 1,000 ms sample array, followed by a 500 ms retention interval, and finally a 3,000 ms test array that was either identical to the sample array (no-change) or varied by a single color (change). Array sizes ranged from 1 to 4 (array size three pictured here), and correct responses were followed by a bell. Feedback was presented at the end of every eight-trial block.

Stimuli for this study were based on Ross-Sheehy and Eschman (2019). Each trial started with a colorful spinning pinwheel that oriented attention, and served as a between-trial mask. Participants were then tested in a change-detection paradigm consisting of a 1,000 ms sample array containing 1–4 colored circles, followed by a 500 ms retention interval, and finally a 3,000 ms test array that was either identical to the sample array (no-change trials) or included a color change presented at a random location (change trials). After 3,000 ms two response buttons appeared underneath the test array, labeled “same” or “different” (Figure 1). Participants saw up to 10 blocks of trials and each block consisted of one of every possible trial type (array size 1, 2, 3, 4, change and no-change) presented randomly.

The circles in both sample and test arrays were presented at 45°, 135°, 225°, and 315° relative to the center of the display, but were constrained to stay within the boundary of the colorful pinwheel perceptual mask. Circles consisted of eight highly discriminable colors (blue, orange, red, yellow, purple, cyan, green and magenta) and were presented against a gray background. Circle locations and colors were chosen randomly without replacement for each trial using a custom python script, and circles for array size 2 were constrained to contiguous locations only (no obliques). Although Gorilla.sc does allow for active stimulus scaling based on visual angle, this scaling operates on individual display objects (i.e., individual circles in our case) and does not address the relative spacing between objects. That is, even though the individual circles might successfully be scaled based on visual angle, the gaps between them were not. Given chunking efficacy might vary with relative circle proximity we chose not to incorporate object-based scaling, and instead opted for passive scaling of the entire configuration based on monitor size. Although this did not explicitly equate visual angle across participants, participants with smaller screens (e.g., laptops or iPads) generally sat closer to the screen, roughly equating visual angle and preserving the relative spaces between the circles.

Engaging sounds were presented during both the sample and test arrays to increase interest in the task, highlight cohesion and alignability between sample and test arrays, and to emphasize the change detection judgment during test array. The sample array sound was an ascending slide whistle that continued through both the sample and gap intervals, followed immediately by a “bloop” sound simultaneous with the onset of the test array. A reward tone immediately followed a correct response, and consisted of a pleasant 630 ms bell tone with a frequency of ~2,300 Hz. There was no feedback given for incorrect trials.

### Procedure

All methods and procedures were approved by University of Tennessee IRB #17-03545. Parents were invited to participate based on previous participation in one of the University of Tennessee Child Development Research Labs. Parents of children 4–10 years were sent an email inviting them to participate in an at-home test of cognitive development. If interested, parents clicked a link, and were taken immediately to an online consent form (children aged 7 and older were additionally assented). Upon completion of the consent, parents filled out a general demographic questionnaire, and were then routed to the online experiment portal (*Gorilla.sc*; Anwyl-Irvine et al., 2020b). Parents and children were given general instructions regarding the online browser-based “memory game,” and informed that the game could be quit and resumed if the child became bored, or if network congestion was high. Parents were then presented with several “get ready” screens, instructing them to ensure their child had a distraction free environment, that their browser was in full screen mode, and that their computer's sound was set at an appropriate level. Prior to online testing, pilot testing occurred in the lab with 3- and 4-year-old children, parents, and adult participants. These experiences helped us determine the youngest feasible age for unsupervised testing, and informed the video demonstration and instructions that appeared prior to the onset of the task. Previous online testing experience suggested this process to be critically important in preventing frustration and enhancing understanding of the task expectations. Parents and children were then presented with a video demonstration of the memory game:

“This colorful pinwheel will appear at the beginning of each trial. Press “Go” to begin. [*child presented with dynamic image of spinning pinwheel and “go” button*]. For each trial, some circles will briefly appear [*child is shown a sample array containing colored circles*], then disappear [*child is shown blank display*], then reappear [*child is shown test array identical to the sample array with the exception of a single color change. After a brief delay, two response buttons were presented underneath the test array, one labeled “Same” one labeled “Different”*]. Your child's job is to determine if the circles stayed the same, or if one of them changed color. Have your child respond aloud, then click “Same” or “Different” to indicate their response. If your child is correct, a bell will ring [*animation of mouse cursor clicking the “Different” button, followed by a bell*]. The circles blink quickly, so be sure not to start the trial until your child is ready! We will vary the position of the circles, and how many appear [*children and parents shown several additional demonstration trials*]. Remember, this was designed to be challenging! If your child is unsure, encourage them to guess.”

Parents and children could watch the video as many times as necessary before proceeding to the practice trials. Practice trials were identical to task trials, however additional instructions were included at the top of each display. Parents clicked “Continue” when their child was ready to begin the task trials. To keep engagement high, children were presented with a performance screen after the completion of each block. This screen provided encouraging feedback, a progress bar, and the child's accuracy. It also included two buttons, one to continue the task trials, and one to end the task early. Parents were instructed to end the trials early if their child became uninterested, or no longer wished to participate. The task took an average of 9.74 min to complete (*SD* = 2.9).

Immediately after task completion, parents and participants were administered a brief survey that included a comment field and two questions assessing enjoyment and comprehension (5-point Likert scale, with one representing least possible enjoyment/understanding, and 5 representing greatest possible enjoyment/understanding). Average ratings for enjoyment (*M* = 3.6, *SD* = 1.17) and task comprehension (*M* = 4.39, *SD* = 1.02) suggested parents and children understood the task, and enjoyed it to a reasonable extent. After participation, parents were emailed a $10 Amazon.com gift card to share with their child.

Two split-half reliability estimates were computed using mean proportion correct at each set size. The first analysis compared accuracy across even and odd trials (i.e., internal consistency) and the second compared accuracy across the first and last half of the trials (i.e., time effects). Cronbach's alpha indicated good internal consistency between even and odd trials, α = 0.712, good reliability over time, α = 0.730. Although mean proportion correct was slightly higher for the first half of the experiment (*M* = 0.888, *SD* = 0.145) compared to the last half of the experiment (*M* = 0.882, *SD* = 0.147), this difference was not significant, *t*_(1, 059)_ = 1.465, *p* = 0.143.

## Results

**Figure 2:**
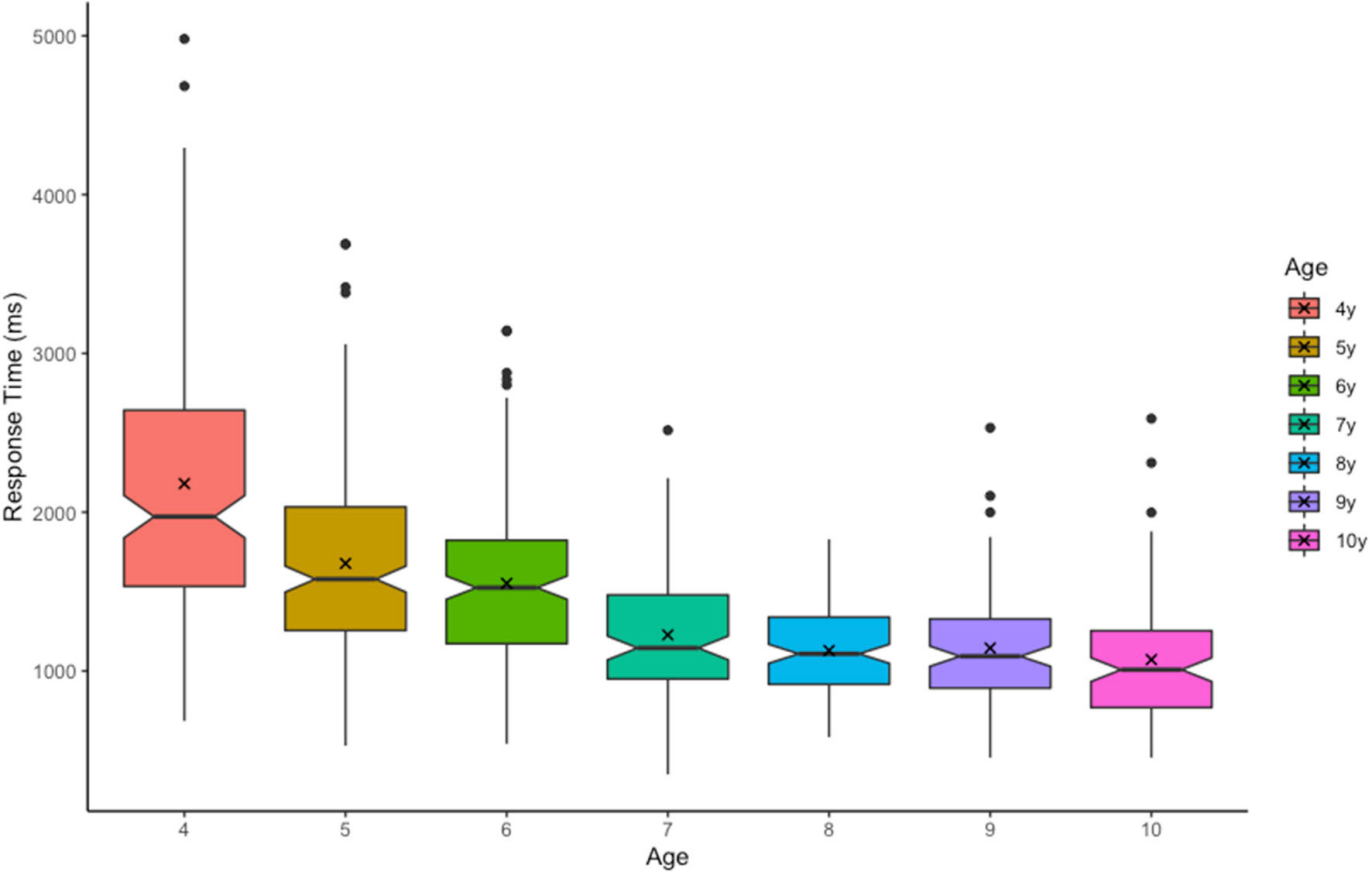
Trial response times (ms) by age. Boxplot edges represent upper and lower quartiles, notches represent the 95% confidence interval of the median (center line), and ‘X' represents the mean.

Raw response times were examined prior to analysis. This revealed one 8-year-old outlier with implausibly high performance (mean response time = 155 ms, perfect performance across all 4 array sizes), who was subsequently removed from our analysis. All other responses conformed to typical developmental patterns (Figure 2). We estimated VWM capacity (*k*) using Pashler's equation (Pashler, 1988) with *k* = *N* x (*H*-*FA*)/(1-*FA*), where *N* = array size, *H* = hit rate (proportion of *change* trials in which color change was correctly detected), and *FA* = false-alarm rate (proportion of *no change* trials in which color change was erroneously detected). We calculated maximum capacity for each child (*max K*) as the highest capacity estimate produced across all four array sizes. Although there is considerable debate regarding the discrete slots assumptions of Pashler's approach (Cowan, 2001; Bays and Husain, 2008; Zhang and Luck, 2008; Rouder et al., 2011), this equation is convenient as it incorporates multiple sources of information and is easier to interpret than accuracy or sensitivity measures such as A' or d'. However, Pashler's equation does not penalize false alarm rates in cases where hit rates were very high. This is one reason why Pashler's equation may slightly *overestimate* capacity, particularly in child samples. For this reason, it is important to prescreen results and identify any participants who may have chosen the same response for every trial. This may also help identify children who were confused by the task.

### Assessing Data Quality, Task Validity, and Environment Variables

#### Does Unsupervised Testing Produce Plausible VWM Capacity Estimates?

**Figure 3:**
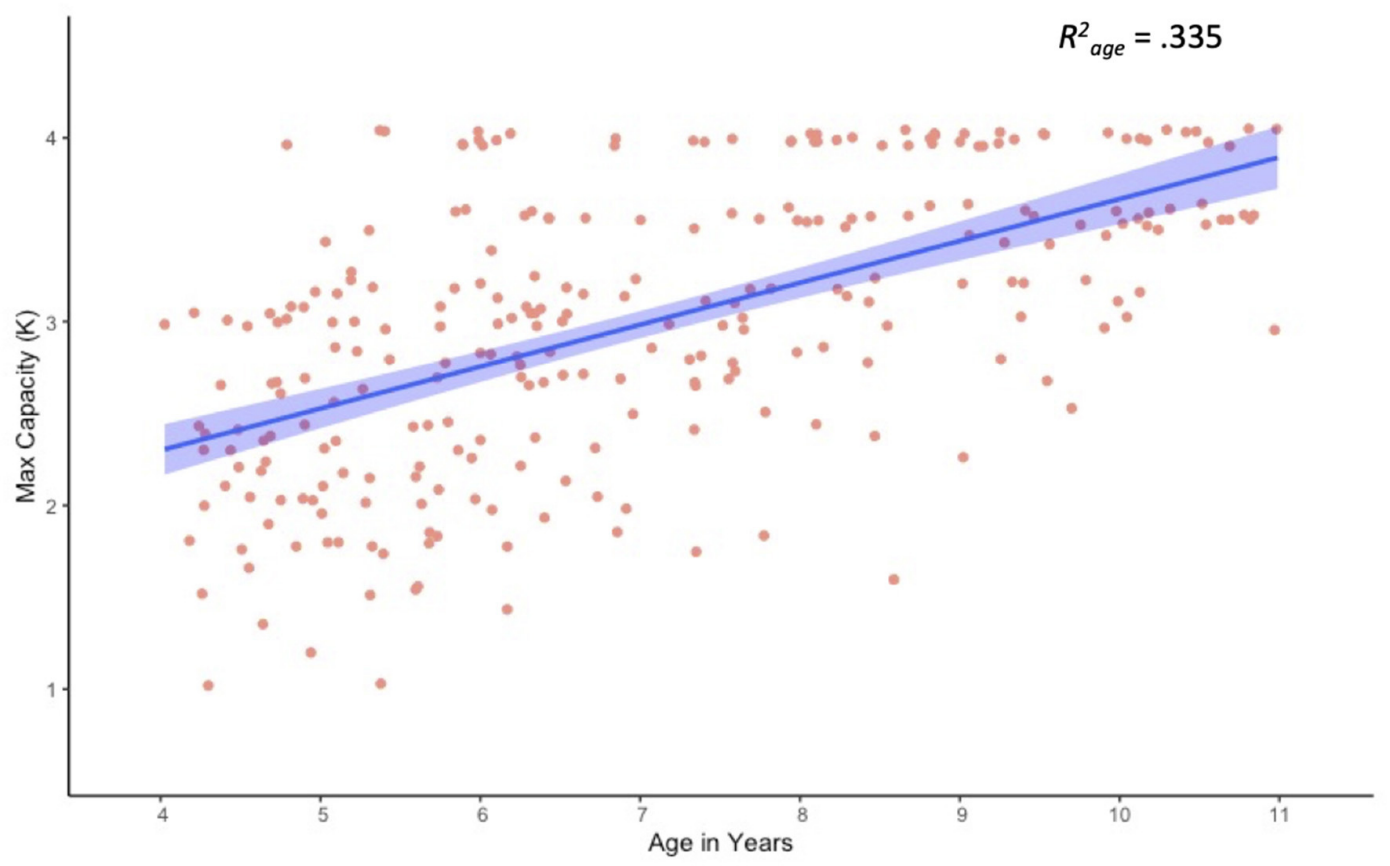
Scatter plot and linear trend for visual working memory capacity (max K) as a function of age.

Because this was an unsupervised task, it was important to assess task performance and compliance, as well as general capacity estimates. A Pearson bivariate correlation revealed a moderate correlation between age and trial counts, *r* = 0.230, *p* < 0.001, with younger children completing fewer trials than older children (Table 2). Although 90.4% of participants completed all 80 trials, the 26 participants who completed fewer than 80 trials were relatively young, *M*_*age*_ = 5.68, *SD*_*age*_ = 1.15. In addition, younger children took longer to respond on average than older children, *r* = −0.565, *p* < 0.001. This finding is not unique to online testing paradigms, and suggests that relatively slow responses may have contributed to increased task fatigue for the youngest children. Importantly, results for maximum capacity (max K) revealed a strong positive correlation with age (Figure 3). These estimates are consistent with previously published findings for children of this age, validating this general approach (Simmering, 2012, 2016; Buss et al., 2018).

**Table 2:** Pearson Bivariate correlation table of task and test environment factors. Significant effects indicated with (^*^).

	**Age**	**Trial count**	**Response time**	**Resolution**	**Response mode**	**Max K**
Age	1	0.230^**^	−0.565^**^	−0.010	−0.105	0.579^**^
Trial Count	0.230^**^	1	−0.311^**^	0.000	0.020	0.059
Response Time	−0.565^**^	−0.311^**^	1	0.120	0.199^**^	−0.419^**^
Resolution	−0.010	0.000	0.120	1	0.409^**^	0.101
Response Mode	−0.105	0.020	0.199^**^	0.409^**^	1	−0.029
Max K	0.579^**^	0.059	−0.419^**^	0.101	−0.029	1

* *p < 0.05*,

** *p < 0.01*,

*** *p < 0.001*.

#### Do Screen Size and Response Mode Influence VWM Capacity Estimates?

One of the drawbacks of at-home testing is the lack of experimental control over the testing equipment and environment (Anwyl-Irvine et al., 2020a). However, there are some important advantages as well. For example, analyzing data collected from home samples facilitates the examination of often ignored task specifics such as the size of the screen (width in pixels), method of response (1 = touchscreen, 2 = keyboard, 3 = mouse) and their influence on VWM capacity estimates. Results of a correlation analysis revealed that neither screen size (*r* = 0.101) nor response mode (*r* = −0.029) were related to VWM capacity, though screen size and response mode were highly correlated, *r* = 0.409, *p* < 0.001 (Table 2). Response mode was also positively correlated with response time (*r* = 0.199, *p* = 0.001), revealing that children responded most quickly when using touchscreen devices (both computers and tablets). Several other significant relations were observed, most notably between response time and max K (*r* = –0.0.419, *p* < 0.001), with faster responding associated with higher capacity estimates, though age may have been an important driver of this effect.

### Assessing Capacity Across Multiple Set Sizes

**Figure 4:**
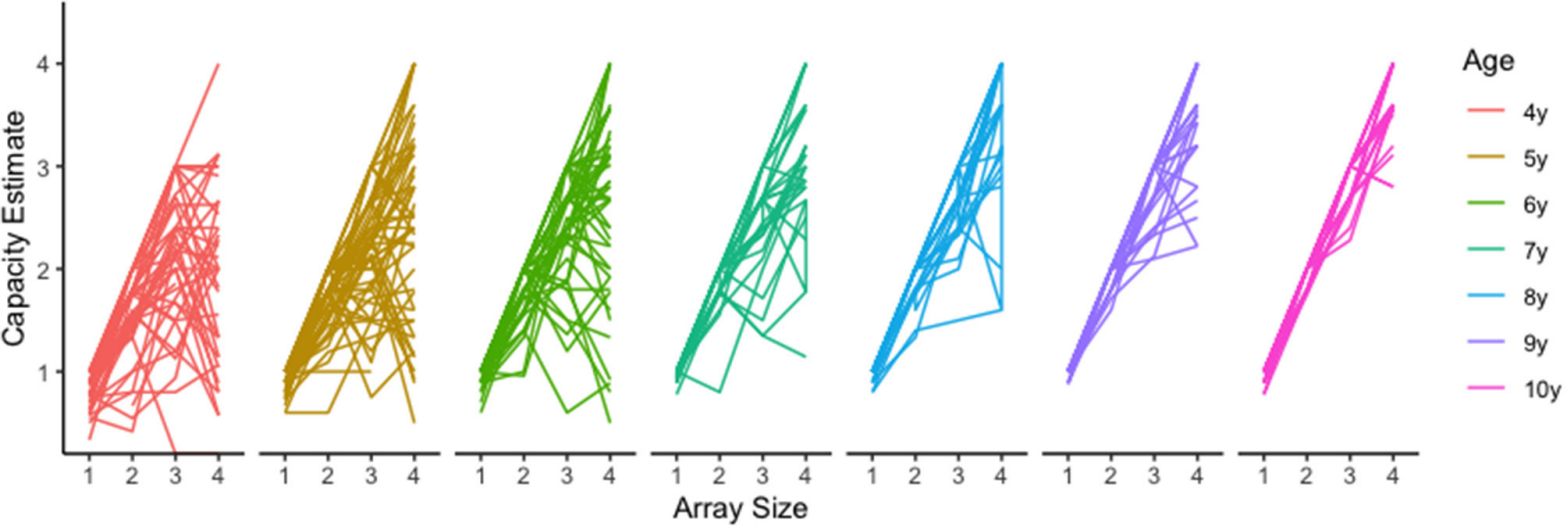
Raw visual working memory capacity trends by age and set size.

**Figure 5:**
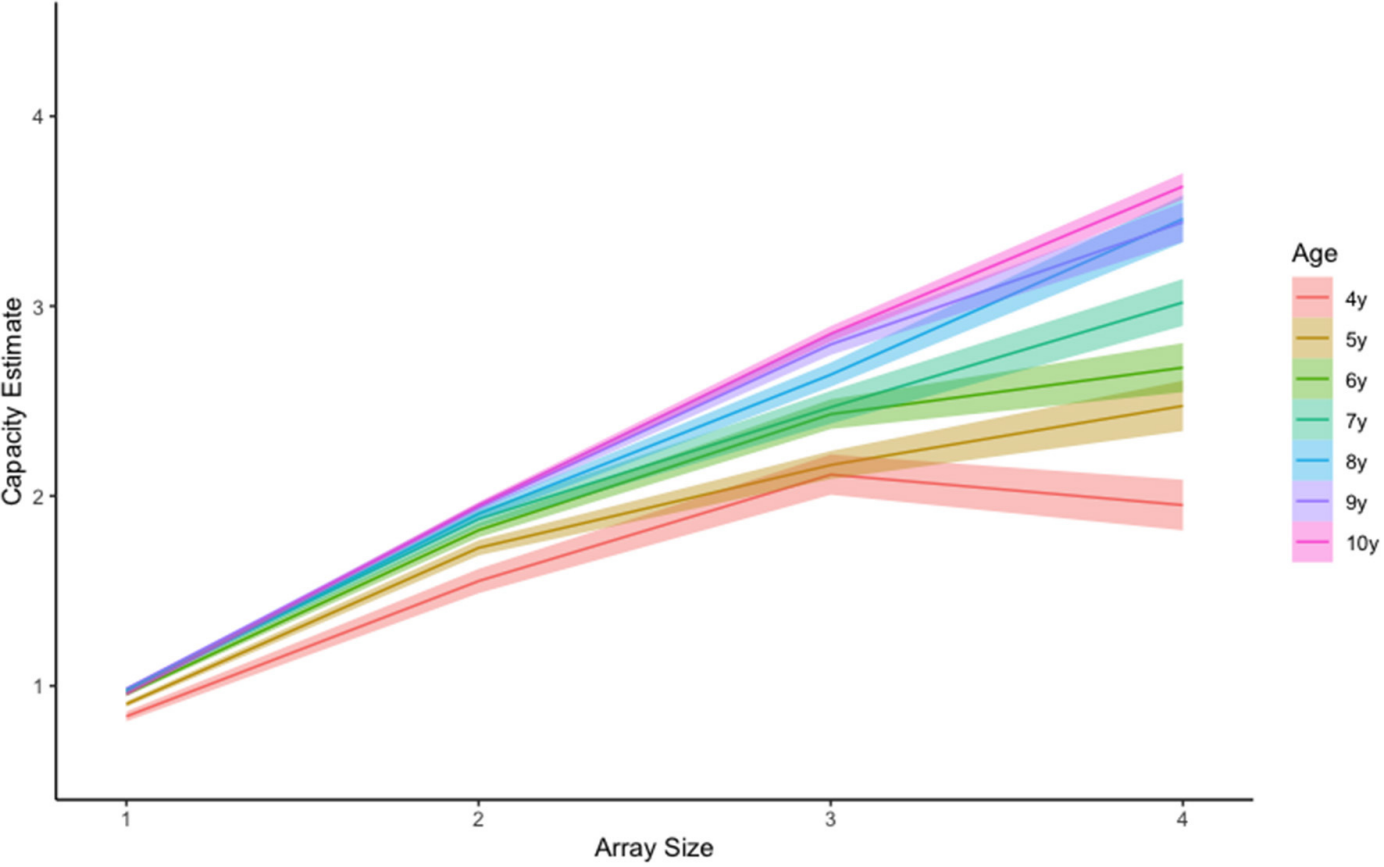
Mean visual working memory capacity trends by age and set size. Shading represents +-1SEM.

Although Pashler's capacity estimate is convenient and easily interpreted, using this equation with child populations poses some unique challenges. One such challenge occurs when hit rates are lower than false alarm rates. In these cases, Pashler's equation will produce a negative value that is uninterpretable. For example, one 5-year-old child in our sample had the following capacity estimates for array sizes 1 through 4, respectively: 1, 1.78, −0.86, and.44. There are two things to notice. First, this child had a negative value for array size 3 (−0.86), however estimates for array sizes 1 and 2 appear valid. Given these negative values were rare (*n* = 9 of 1,051 cells) we treated them as missing data and removed them from the analysis. The second thing to notice, is that the capacity estimate for array size 4 is *smaller* than estimates for array size 2 and even array size 1. We believe this may occur when children become overwhelmed by the memory demands for a given array and disengage from the task. There is some neurophysiological evidence to support this (Fukuda et al., 2010; Reyes et al., 2020; McKay et al., 2021). If this is the case, then the array size that produces maximum capacity (i.e., the *optimal array size*) should vary by age, with younger children reaching maximum capacity for smaller array sizes, and older children reaching maximum capacity for large array sizes independent of capacity estimates. An examination of the raw data clearly reveals such a trend (Figure 4), with younger children showing apparent capacity regressions at higher array sizes (Figure 5).

#### Do Large Arrays Disproportionately Hinder VWM Performance for Younger Children?

To determine if large array sizes resulted in underestimation of capacity for our young participants, we conducted a linear mixed effect (LME) analysis using R (R Core Team, 2020) with package lme4 (Bates et al., 2015). LME analyses are robust to missing data, and can handle the interdependence of capacity estimates across array size (Singmann and Kellen, 2019). This approach allowed us to calculate the extent to which capacity estimates increased with increasing array size for each age. We included fixed effects of array size and age and random participant-level effects in our baseline model (i.e., random intercept). Based on the observation that capacity varied with age (Figure 4), we additionally included an array size by age interaction. This addition significantly improved model fits, χ^2^ (18, *N* = 1,051) = 199.93, *p* < 0.001.

Effect estimates from our full LME model are presented in Table 3, and estimated marginal means are presented in Table 4. Age and array size were dummy coded so that the intercept reflects mean capacity for our reference group (4-year-olds at array size 1), and estimates reflect deviations from reference. Results for age were not significant, suggesting that despite small differences in array size 1 estimates (e.g., K = 0.83 at 4 years versus K = 0.96 at 10 years) all ages performed at ceiling for array size 1. However, results for array sizes 2–4 varied markedly by age. For example, though all ages had significant array size 4 effects, only 8- to 10-year-olds demonstrated significant array size 3 effects, with only 9- and 10-year-olds showing additional marginal effects for array size 2. This makes sense, as the slope of the regression line for array size should increase as overall capacity estimates increase (Figure 3).

**Table 3:** Estimates and model fits for predictors of visual working memory capacity. Significant effects indicated with (^*^).

**Model**	**Source**	**Estimates**	**SE**	**df**	***t***	***p***
Full model	Intercept	0.834	0.075	926.188	11.168	<0.001^***^
	Array size 2	0.717	0.092	791.429	7.754	<0.001
	Array size 3	1.276	0.094	797.340	13.611	<0.001^***^
	Array size 4	1.115	0.094	797.340	11.898	<0.001^***^
	5y	0.068	0.099	939.852	0.688	0.492
	6y	0.123	0.102	914.485	1.206	0.228
	7y	0.149	0.115	902.258	1.294	0.196
	8y	0.141	0.116	892.237	1.222	0.222
	9y	0.150	0.117	911.148	1.282	0.200
	10y	0.124	0.121	910.477	1.026	0.305
	Array size 2 ^*^5y	0.106	0.123	790.210	0.866	0.387
	Array size 3 ^*^5y	−0.022	0.124	794.534	−0.176	0.861
	Array size 4 ^*^5y	0.450	0.124	796.411	3.619	<0.001^***^
	Array size 2 ^*^6y	0.146	0.126	790.117	1.154	0.249
	Array size 3 ^*^6y	0.198	0.127	793.346	1.556	0.120
	Array size 4 ^*^6y	0.604	0.127	793.346	4.746	<0.001^***^
	Array size 2 ^*^7y	0.183	0.142	789.795	1.285	0.199
	Array size 3 ^*^7y	0.213	0.143	792.350	1.486	0.138
	Array size 4 ^*^7y	0.925	0.143	792.350	6.466	<0.001^***^
	Array size 2 ^*^8y	0.219	0.142	789.795	1.539	0.124
	Array size 3 ^*^8y	0.393	0.143	792.350	2.747	0.006^**^
	Array size 4 ^*^8y	1.374	0.143	792.350	9.604	<0.001^***^
	Array size 2 ^*^9y	0.241	0.145	789.749	1.659	0.098
	Array size 3 ^*^9y	0.538	0.146	792.208	3.688	<0.001^***^
	Array size 4 ^*^9y	1.343	0.146	792.208	9.204	<0.001^***^
	Array size 2 ^*^10y	0.276	0.150	789.676	1.838	0.066
	Array size 3 ^*^10y	0.620	0.151	791.979	4.112	<0.001^***^
	Array size 4 ^*^10y	1.557	0.151	791.979	10.329	<0.001^***^
	**AIC**	**BIC**	**LogLik**	**Chisq**	**df**	***p***
Baseline model	1618.60	1678.10	−797.30	–	–	–
Full model	1454.70	1603.40	−697.34	199.930	18.00	<0.001^***^

* *p < 0.05*,

** *p < 0.01*,

*** *p < 0.001*.

**Table 4:** Estimated marginal means based on best-fitting LME model (full model).

**Age**	**Set size**	**Mean**	**SE**	**df**	**Lower CI**	**Upper CI**
4 years	1	0.834	0.076	953	0.686	0.983
	2	1.551	0.075	944	1.404	1.698
	3	2.110	0.077	961	1.960	2.260
	4	1.949	0.077	961	1.799	2.100
5 years	1	0.902	0.066	940	0.773	1.031
	2	1.725	0.066	940	1.596	1.854
	3	2.156	0.066	947	2.026	2.286
	4	2.467	0.067	960	2.335	2.599
6 years	1	0.957	0.070	927	0.819	1.095
	2	1.820	0.070	927	1.682	1.958
	3	2.431	0.070	927	2.293	2.569
	4	2.676	0.070	927	2.538	2.814
7 years	1	0.983	0.089	910	0.809	1.158
	2	1.883	0.089	910	1.708	2.057
	3	2.472	0.089	910	2.297	2.646
	4	3.024	0.089	910	2.849	3.198
8 years	1	0.976	0.089	892	0.800	1.151
	2	1.911	0.089	892	1.736	2.087
	3	2.644	0.089	892	2.469	2.820
	4	3.465	0.089	892	3.289	3.640
9 years	1	0.985	0.091	927	0.805	1.164
	2	1.942	0.091	927	1.762	2.121
	3	2.798	0.091	927	2.619	2.978
	4	3.442	0.091	927	3.263	3.622
10 years	1	0.959	0.097	927	0.769	1.148
	2	1.951	0.097	927	1.761	2.141
	3	2.854	0.097	927	2.665	3.044
	4	3.631	0.097	927	3.441	3.820

To assess these patterns more directly, we conducted follow-up contrast analyses for each age (R package: emmeans v1.5.5-1) using estimated marginal means derived from our LME model (Searle et al., 1980). Significant non-linear trends would suggest that capacity estimates peaked for smaller array sizes, then regressed for larger array sizes. Results revealed significant quadratic trends for our four youngest ages: 4 years, *t*_(820)_ = −6.551, *p* < 0.001, 5 years, *t*_(817)_ = −4.389, *p* < 0.001, 6 years, *t*_(812)_ = −5.007, *p* < 001 and 7 years, *t*_(812)_ = −2.242, *p* = 0.025. These findings highlight 4–7 years as an ideal age at which to identify and track individual differences, and underline the importance of including smaller array sizes to catch maximum capacity performance for younger children. Although we see a great deal of variability in our youngest participants, performance for 8-, 9-, and 10-year-olds did not appear to differ. This observation coupled with relatively large capacity estimates for these older children, suggests that VWM capacity improvements may have slowed by 8-years-of age, approaching adult capacity of around 3–4 items (Rouder et al., 2011; Zhang and Luck, 2011).

## Discussion

Children ages four through 10 were tested in an unsupervised, online change-detection task. Results from this paper highlight several novel benefits of online testing. For example, online approaches are quick, have compliance rates comparable to lab-based techniques, and appear to provide accurate results on par with lab-based approaches. In addition, online testing may increase diversity of the sample, facilitate testing across a wide array of ages, and allow for testing across regions, or even countries. Other benefits of this approach include reduced resource and infrastructure demands, increased testing speed (~300 participants tested around 6 months vs. 2–3 years for in-lab testing), and the ability to allow maximum flexibility for parents and children, so that sessions may be timed when participants are maximally attentive.

Although there are several challenges to testing online, we did not find them to be unsurmountable. For example, ensuring that participants (not the parents) completed the assessments could be handled by capturing periodic facial images during testing, something that is possible with most browser-based experimental software suites. This may be particularly important if the task is being advertised broadly and compensation is provided. Although we did not collect participant video in our sample, we limited participation to families in our local area with whom we had a prior relationship, either as participants in our own lab or in our departmental colleagues' labs. In addition, pre-screening the data prior to analysis can help identify suspicious data (e.g., response times too quick or performance too high). All tasks should be piloted in-lab to help develop expectations for performance, and to identify any issues with the task, or with child and/or parent understanding of the task.

Our results revealed several insights regarding at-home testing, such as the importance of tracking as many environment variables as possible. Although we found no evidence that screen size and response mode impacted VWM capacity estimates, it is possible that exceptionally large or small screens might still be problematic. We did find evidence that response mode influenced the speed of responding, which might be an issue for speeded designs or designs that require some sort of response inhibition (e.g., flanker or go/no go tasks). Some of our findings were not unique to online testing, such as the finding of slower response times for younger kids, and larger arrays sizes (older children only).

In addition to demonstrating the validity of unsupervised online testing approaches, our results also produced several novel insights regarding the development of VWM from 4 through 10 years of age. First, our results produced capacity estimates that are comparable to published lab-based estimates (Cowan et al., 2005; Riggs et al., 2006; Simmering, 2016), suggesting this approach to be a viable alternative requiring a fraction of the resources necessary for lab-based tasks. In addition, we found capacity increased significantly with age, reaching near-adult levels by around 8-years-of age (Figure 3). Our analysis also revealed evidence of substantial performance variability from 4- to 7-years-of-age (Figure 4), potentially highlighting assessment points for longer-term individual difference studies, as well as possible targets for memory intervention. Given the ease of online testing and the importance of VWM to several aspects of math and cognitive performance (Jarvis and Gathercole, 2003; Bull, 2008; Tsubomi and Watanabe, 2017; Giofrè et al., 2018; Allen et al., 2019; Chan and Wong, 2019; Kyttälä et al., 2019; Carr et al., 2020), adding a quick at-home assessment as part of a school, medical, or lab assessment might provide a more detailed developmental profile.

### On Estimating Capacity in Children

One of our most important findings was the demonstration of an interaction between array size and capacity estimation, especially for our youngest participants. Whereas, our older participants appeared able to perform consistently regardless of array size, our youngest participants seemed to disengage for larger arrays, resulting in estimates that were often lower than estimates obtained from smaller arrays. This is evidenced visually in our raw data (Figure 4), and statistically in our finding of significant quadratic trends for our 4- through 7-year-olds. These errors may have been purposeful (i.e., sample array perceived as too difficult resulting in a random guessing strategy), or they may have occurred after earnest attempts to respond accurately. If an explicit guessing strategy was employed for larger array sizes, we would expect mean response times to be negatively correlated with array size. A correlation analysis on the raw data revealed this may be the case, with 4- through 7-year-olds demonstrating a small but significant *negative* correlation between response time and array size, *r* = −0.061, *p* = 0.031, and 8- through 10-year-olds revealing a small but significant *positive* correlation, *r* = 0.093, *p* = 0.032.

The finding of slightly faster response times for large array sizes suggests that at least some of our youngest participants may have resorted to guessing strategies when the demands of the array exceeded memory capacity, attentional resources, or some combination of the two. This is consistent with previous work demonstrating that children have sufficient metacognitive awareness to know when they have successfully encoded a to-be-remembered event, and when they have not (Applin and Kibbe, 2020). However, it is also possible that this drop in performance for set size 4 arrays may be the result of *catastrophic forgetting*, or the inability to encode any array items when capacity is exceeded. For example, in manual search tasks, 12- and 14-month-old infants appear unable to detect the difference between hiding events involving two vs. four balls, despite successfully detecting the difference between two vs. three balls (Feigenson and Carey, 2003). Importantly, this effect may have been partially driven by perceptual similarity, as it is largely ameliorated when four differently colored balls are used (Zosh and Feigenson, 2012). Given the older participant ages tested here and our use of highly discernably circle colors, it seems unlikely that the drop in performance for large arrays is the result of catastrophic forgetting.

Although adult researchers have proposed avoiding small array sizes to reduce the likelihood of underestimation (Morey, 2011), our results suggest that using large array sizes might also underestimate capacity, particularly for our youngest participants. Without a doubt, probabilistic and Bayesian approaches to capacity estimation are more sophisticated and can better account for high false alarm rates present in our young samples. However, these analysis techniques are not as readily adapted to online calculation or quick assessment for individual participants. We believe using a variety of array sizes works well as long as assessments are based on either *maximum* capacity across array sizes, or a holistic assessment of capacity as a *function of array size*. It is possible that reducing the number of large array sizes would increase number of trials young children complete, but those benefits would have to be weighed against the possible cost of underestimating capacity due to ceiling effects for higher performing children. If the goal of the assessment is to identify general working memory ability, a more desirable metric might be the *array size* at which a child reaches maximum capacity, or the *optimal array size*. This metric incorporates both a *quantitative* capacity estimate (i.e., maximum capacity) and a *qualitative* attentional estimate (i.e., maximum array size a child can tolerate before disengagement).

In conclusion, results presented here demonstrate the feasibility of effective and accurate at-home assessments of VWM, and provide novel insights into the influence of factors such as array size, screen size, and response mode. Results additionally highlight numerous benefits for unsupervised at-home testing, from substantially increasing sample diversity (e.g., SES, race, ethnicity) to enabling large-scale geographically unconstrained population surveys at a relatively low cost. We have also found that allowing participants the flexibility to pick optimal test times increases compliance, decreases stress, and contributes to improved data quality and representativeness. Although this approach may not be useful for tasks that require closely monitored speeded approaches, it seems quite appropriate for change-detection tasks. Future work will be conducted to test older ages and broaden our participant pool geographically to include underrepresented regions and populations. It is our hope that approaches like the one presented here may help identify regional, cultural, and socioeconomic influences that affect VWM development and general cognitive outcomes.

## Data Availability Statement

The raw data supporting the conclusions of this article are available for download at https://osf.io/2b8zg/?view_only=44f50ae4514d415c8da887c53431fd14.

## Ethics Statement

The studies involving human participants were reviewed and approved by University of Tennessee IRB #17-03545. Written informed consent to participate in this study was provided by the participants' legal guardian/next of kin.

## Author Contributions

SR-S, ER, and BE contributed to conception and design of the study. SR-S and ER performed the statistical analysis. SR-S wrote the first draft of the manuscript. All authors contributed to manuscript revision, read, and approved the submitted version.

## Conflict of Interest

The authors declare that the research was conducted in the absence of any commercial or financial relationships that could be construed as a potential conflict of interest.

## Publisher's Note

All claims expressed in this article are solely those of the authors and do not necessarily represent those of their affiliated organizations, or those of the publisher, the editors and the reviewers. Any product that may be evaluated in this article, or claim that may be made by its manufacturer, is not guaranteed or endorsed by the publisher.
